# A retrospective study of the efficacy of intense pulsed light delivered by the Lacrystim® for meibomian gland dysfunction therapy

**DOI:** 10.1186/s12886-022-02531-7

**Published:** 2022-08-06

**Authors:** Marie-Caroline Trone, Thibaud Garcin, Edouard Ollier, Gilles Thuret, Philippe Gain

**Affiliations:** 1 Ophthalmology department, University Hospital, 42055 Saint-Etienne Cedex1, Saint-Etienne, France; 2 Corneal Graft Biology, Engineering and Imaging Laboratory BiiGC, EA2521, Health innovation campus, faculty of Medicine, Jean Monnet University, Saint-Etienne, France; 3 Clinical Research, Innovation and Pharmacology Unit, University Hospital, Saint-Etienne, France; 4 Health engineering biology (SAINBIOSE) Inserm U1059, vascular hemostasis dysfunction (DVH) team, Health innovation campus, faculty of Medicine, Jean Monnet University, Saint-Etienne, France

**Keywords:** Intense pulsed light, Meibomian gland dysfunction, NIBUT, Dry eye disease

## Abstract

**Background:**

Meibomian gland dysfunction is the most common etiology of dry eye disease worldwide and intense pulsed light appears to be a promising treatment with encouraging results. Lacrystim® is a new IPL device (CE marking in 2019) and no studies have yet been published on it. We propose the first study on this device with an objective assessment of its efficacy and an extended follow-up over 6 months.

**Methods:**

Patients presenting with a dry eye disease (DED) with stable mild to moderate MGD and having received Lacrystim® treatment between june 2019 and june 2020 were included. 3 IPL sessions were performed at D0, D15 and D45 with 4 shots per side at a fluence of 8 mJ/cm2. DED clinical evaluation was performed at D0, D15, D45, 3rd month and 6th month: Oxford scale and break up time, Schirmer test and Ocular Surface Disease Index (OSDI) questionnaire. Lacrydiag® imaging device carried out an objective examination of tear film: interferometry, meibography, tear meniscus height and non-invasive break up time (NIBUT). The primary endpoint was the evolution in NIBUT between the first visit D0 and 3rd month. Data collection was done retrospectively. Statistical analysis was done using a linear mixed-effects model and a non-parametric linear mixed-effects model (R software).

**Results:**

Forthy five consecutive patients were included. NIBUT significantly increased between D0 and 3rd month: mean difference of 1.63 seconds, IC95% [0.51; 2.62], (*p =* 0.002) with a prolonged effect at 6th month. OSDI and OXFORD scores and interferometry were also significantly improved at 3rd month and 6th month. There was no significant change in BUT, Schirmer test and tear meniscus height. No adverse event was noted.

**Conclusions:**

IPL delivered by Lacrystim® appears effective and safe to treat MGD although a randomized controlled trial is needed to validate its results.

**Trial registration:**

This work was approved by a local ethics committee “Terre d’éthique” (institutional review board number: IRBN672019/CHUSTE) and registered on the clinicaltrial.gov website (NCT04147962, 01/11/2019).

## Introduction

Meibomian gland dysfunction (MGD) is the most common etiology of dry eye disease (DED) globally. It causes evaporative dry eye symptoms [1–3] combining eye irritation, tear film alteration and chronic inflammation with a functional disability that may be significant. MGD treatment is based a number of principles which can be used either alone or in conjunction: artificial tears, eyelid hygiene (warm compresses, massage, gland expression), lid debridement, blink rehabilitation, wet chamber warming goggles, azithromycin cures or other anti-inflammatory eye drops [4].

Polychromatic intense pulsed light (IPL) has historically been used in dermatology for the treatment of various cutaneous conditions including vascular and pigmented lesions, tattoos, scars, undesired hair, rejuvenation, photodynamic photorejuvenation, carcinology, rosacea, inflammatory and retentional acne [5, 6].

More recently IPL has been proposed in ophthalmology as an alternative treatment of MGD (step 2 in TFOS DEWS II management algorith [7] with promising results on tear film quality [8–10]. Currently, five IPL devices are available on the ophthalmology market: E-Eye® (E-Swin, Houdan, France), M22 Optima IPL® (Lumenis, Borehamwood, UK), Eye-Light® (Topcon, Tokyo, Japan), Thermaeye® (MSD, Madrid, Spain) and Lacrystim® (Quantel medical, Clermont-Ferrand, France). Although its mechanism of action is not completely elucidated, several theories are currently being studied [11, 12]: stimulation of parasympathetic innervation, acceleration of meibomius glands metabolism, better expression of the meibum under the effect of heat, coagulation of small vessels reducing skin and eyelid inflammation, and reduction of demodex [13].

Initial studies on IPL application for the treatment of MGD used subjective criteria such as OSDI score or break-up-time (BUT) to determine their effectiveness.

To this effect, objective image analysis criteria is used to evaluate IPL efficacy.

The objective of our study is to conduct a retrospective chart review of patients who have been treated with IPL using the Lacrystim®.

## Methods

### Study design

This study was a retrospective, non-randomized observational and monocentric study. It followed the tenets of the Declaration of Helsinki. Patients were involved in the conduct, reporting and dissemination plans of this work. As this was a retrospective study, they were all informed by email and/or phone of the use of their medical data for scientific and publication purposes by mail. This work was approved by a local ethics committee “Terre d’éthique” (institutional review board number: IRBN672019/CHUSTE) and registered on the clinicaltrial.gov website (NCT04147962, 01/11/2019).

### Objectives and end points

The main objective of this work was to measure the three-month IPL efficacy in a series of consecutive cases of MGD using objective criteria provided by image analysis of the ocular surface. The secondary objective was to assess IPL safety in this indication. The end point was the evolution of non-invasive break-up time (NIBUT) between the first visit (D0) and 3 months (3rd month). NIBUT is a marker of tear film homeostasis and is one of the diagnostic tests recommended by DEWS II for the diagnosis of DED [14]. Longer-term results (6th month) were also analyzed. Safety of IPL treatment was assessed by collecting all adverse events. Data collection was done retrospectively from patients’ medical records. It concerned: demographic data (sex, age), ocular surface characteristics (see below), treatment parameters, presence or absence of adverse events (burns, tingling, skin redness). To avoid selection bias, all consecutive patients treated in the ophthalmology department of the university Hospital of Saint-Etienne between June 2019 and June 2020 were analyzed.

### Patients

Adults presenting with a DED with stable and symptomatic MGD [15] for at least 6 months and having received treatment with Lacrystim® in the ophthalmology department of the university Hospital of Saint-Etienne between June 2019 and June 2020 were included. Definition and diagnosis of MGD were made according to the criteria defined by The International Workshop on Meibomian Gland Dysfunction report. Symptomatic MGD is defined by an MGD with both subjective and objective features. The key signs of MGD are as follows: meibomian gland dropout, altered meibomian gland secretion, and changes in lid morphology [15]. Exclusion criteria were limited to the presence of skin diseases contraindicating IPL (active skin infection, photosensitizing drugs, keloid scars, dermabrasions or pigmented lesions in the treatment area) and modification of systemic or local concomitant therapies, including those for MGD during the 6 months before and during treatment (artificial tears, eyelid massages, ciclosporin eyedrops).

### IPL treatment by Lacrystim®

**Fig. 1:**
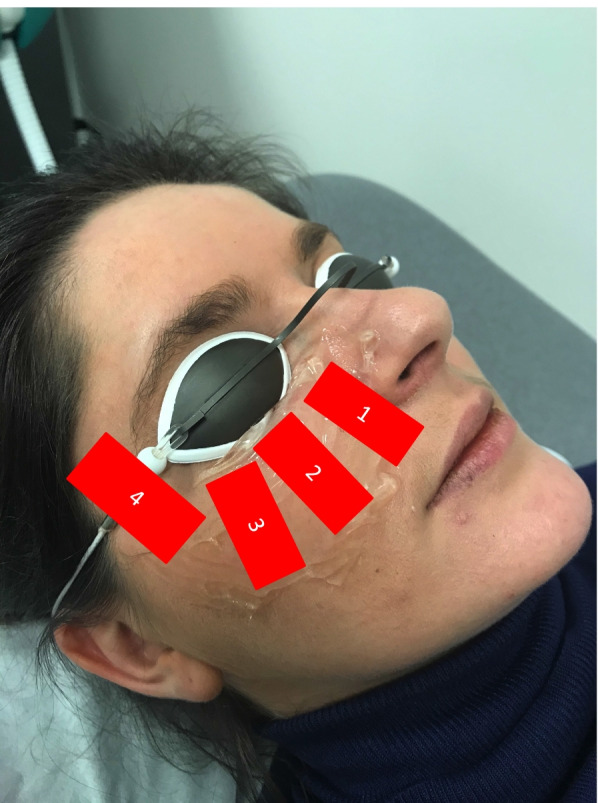
Installation during a session of IPL treatment. The patient closed his/her eyes under the protective glasses during shooting. A is applied to the cheekbones. Four shots are made per side. (publication permission obtained from the patient)

The Lacrystim® is a CE marked IPL device emitting polychromatic light with a wavelength spectrum from 610 to 1200 nm. This spectrum reduction to 610 nm is possible thanks to a filter at 610 nm limiting ultraviolet rays and allowing less absorption by melanin and therefore suitable for the treatment of all skin phototypes, including IV, V and VI. The fluence comprised between 8 and 12 mJ/cm^2^ is delivered by a train of pulses, in order to reduce tissue heating and consequently inflammatory reaction and pain. Treatment was carried out according to the usual protocol in 3 sessions: at day D0, D15 and D45. Each treatment session consisted of four shots on each face side (3 on the cheekbone and 1 on the temple) at a fluence of 8 mJ/cm^2^. The handpiece was applied onto the skin using a thin layer of transparent gel (Gel-Larmes, Thea, Clermont-Ferrand, France). Nevi and tattoos were protected by strips and protective glasses were worn by both practitioner and patient (Fig. 1). At the end of the session, a moisturizing cream (vitamine A Dulcis, Allergan, Dublin, Irlande) was applied on the patient’s face and he/she was advised not to expose him/herself to the sun for the next 24 hours.

### Ocular surface assessment

The ocular surface of both eyes was assessed using the Lacrydiag® imaging device (Quantel Medical, Clermont-Ferrand, France) that provided analysis of the different layers of the tear film: interferometry for the lipid layer (using Guillon classification [16], height of the tear meniscus for the aqueous layer and NIBUT for the mucinic layer. NIBUT obtained by dynamic recording of tears (tear film breaks) after projection on the cornea of placido disc. In addition, the meibomius glands were analyzed by infrared imaging of the eyelids. Images acquisition was independent of observer’s skill. NIBUT acquisition and analyses is fully automated, making NIBUT an objective criterion. Images acquisition of interferometry is automatic but its analysis is manual (choice of an interferometry stage by image comparison). Height of tear meniscus is realized from an image by manually placing calipers. For meibography, upper eyelids are inverted and the contour of the analysis area is manually drawn. The percentage of meibomius glands loss is automatically detected.

In addition to the examination with Lacrydiag® device, we performed a standard slit-lamp examination using fluorescein instillation to determine the Oxford scale and BUT, a Schirmer test without anaesthesia for 5 min, and the ocular surface disease index (OSDI) was calculated using the standardized questionnaire [17, 18].

This ophthalmological examination was performed at D0, just before IPL treatment, and repeated at D45, month 3rd month and 6th month, that is to say immediately after and then 1.5 and 4.5 months after the last IPL session.

### Statistical analysis

Ocular surface assessment data were analyzed using a linear mixed-effects model with random intercept to account for their longitudinal nature. To account for both between-patient and between-eye (left eye or right eye) variabilities nested random effects were implemented. In case of data with a non-Gaussian distribution a non-parametric linear mixed-effects model [19] was used. Boxplot graphs are presented. The criterion for statistical significance was *p* < 0.05. Data analysis was performed using the R software [20]. All graphics were generated using the ggplot2 softwacre package [21].

## Results

### Baseline characteristics of patients

Forty-five patients (90 eyes) were included. Average age was 59, 9 years old (26; 80) with a sex ratio of 1/3 in favor of women (15 men and 30 women). All of them had acquired MGD. 6 patients had MGD with hyposecretory status (iatrogenic origin). 39 had MGD with obstructive status: 5 were cicatricial (graft-versus-host disease) and 34 were non cicatricial (27 primary and 7 secondary with 5 atopies and 2 seborrheic dermatitis). Patients phototypes were determined using the Fitzpatrick classification [22] (phototype I:0, II: 3, IV: 24, V: 16, VI: 0). MGD severity was scored with individual patient parameters [15] (stages 1 to 4): 24 patients had a stage 2, 19 patients with stage 3 and only 2 patients had a stage 4.

Their baseline ocular assessment was summarized (Table 1). Eight patients were lost of follow-up at 6th month due to the COVID-19 outbreak and confinement.

**Table 1:** Baseline characteristics of the ocular surface before treatment with intense pulsed light

	NIBUT (seconds)	OSDI	OXFORD	BUT (seconds)	SCHIRMER (mm)	Interferometry	***Tear meniscus height (mm)***
Number of patients	90	45	90	90	90	90	90
Mean (standard deviation)	8.9 (3.0)	44.2 (20.6)	1.6 (1.2)	15.6 (7.5)	14.7 (12.1)	43.1 (28.8)	0.3 (0.2)
Median	8.6	43.8	2.0	15.0	11.0	30.0	0.2
Q1,Q3	7.4, 11.1	29.2, 56.8	1.0, 2.0	10.0, 20.0	5, 27.0	15.0, 80.0	0.2, 0.4
Range	3.4–15.6	0.0–97.9	0.0–5.0	3.0–45.0	0.0–35.0	12.0–120.0	0.1–1.1

### The non-invasive break-up-time, OSDI, Oxford score and interferometry improve after IPL (Table 1)

**Fig. 2:**
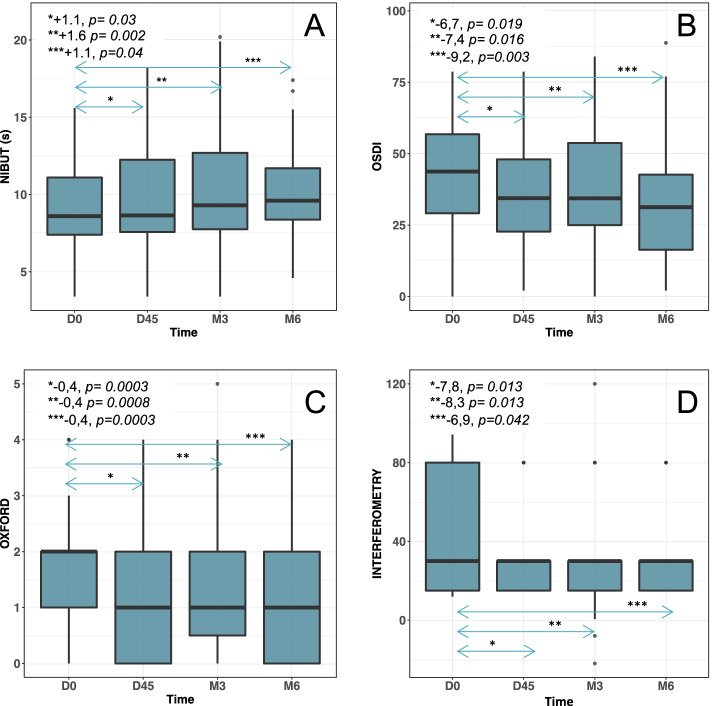
Boxplot of non-invasive break-up-time (NIBUT) (A), ocular surface disease index (OSDI) (B) Oxford score (C) and interferometry (D) changes over time. Thick horizontal lines show the distribution median; boxes, the interquartile range (IQR); and individual points, the outliers between 1.5 and 3 times the IQR. Whiskers mark the highest and lowest non-outlying values. **A** Median NIBUT increased with time: 8.6 at day (D) 0, 8.7 at D45, 9.3 at 3rd month and 9.6 at 6th month. **B** OSDI score significantly improved over time with medians at 42.7 at D0, 34.2 at D45, 34.3 at 3rd month and 35.4 at 6th month. **C** Oxford score improved over time with medians at 2.0 at D0, 1.0 at D45, 1.0 at 3rd month and 1.0 at 6th month. **D** Interferometry significantly improved with a constant median over time at 30 at D0, D45, 3rd month and 6th month

The mean difference in NIBUT between D0 and 3rd month (primary endpoint) was 1.6 seconds CI95% = [0.5; 2.6] with a NIBUT mean value at 3rd month of 10.5 seconds. NIBUT values at D45 and 6th month were also statistically improved. The difference in NIBUT between D0 and D45 was 1.1 seconds 95%CI = [0.1; 2.1] and 1.1 seconds _95%_CI = [0.1; 2.2] between D0 and 6th month (Fig. 2A).

The *p*-values of the likelihood ratio test and the non-parametric test were equal to 0.016 and 0.009 respectively, so the time at which the visit was conducted had a statistically significant influence on the NIBUT value. The greatest improvment in NIBUT was at 3rd month.

**Fig. 3:**
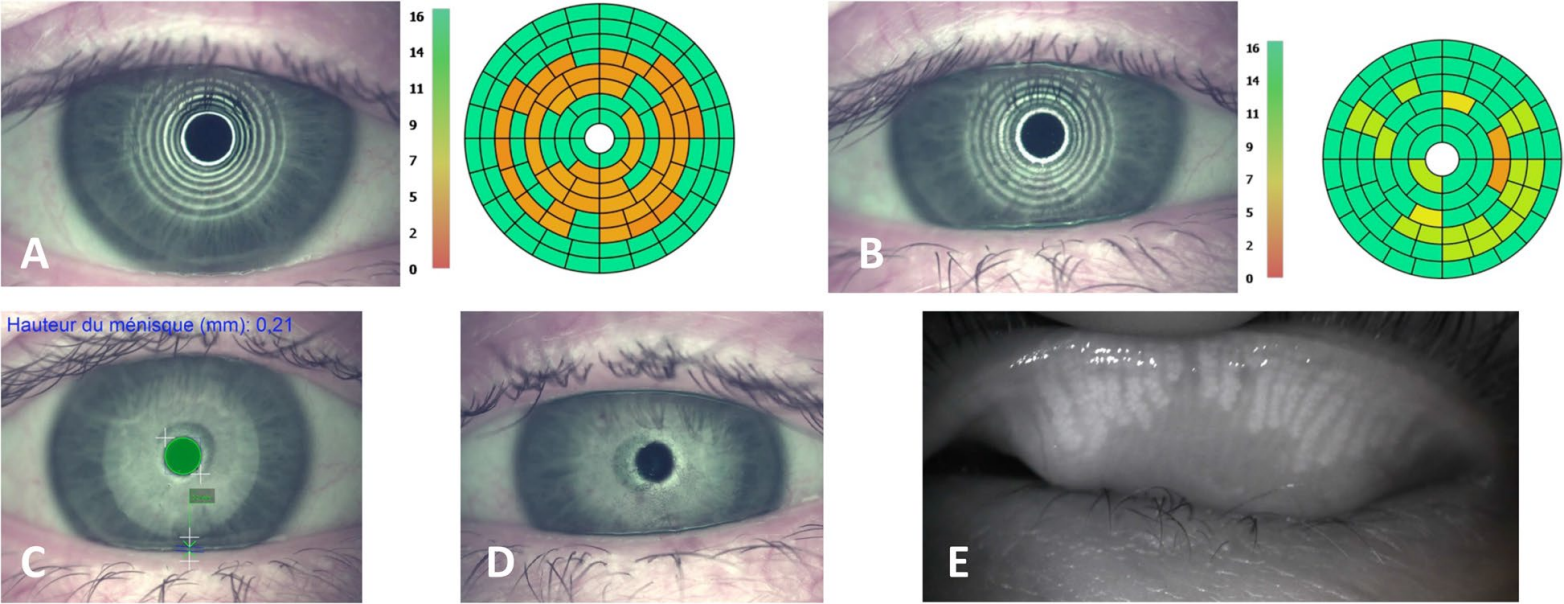
Example of tests performed with LACRYDIAG® on a patient whose OSDI score improves from 45.8 at D0 to 27.08 at 3rd month. **A** NIBUT at D0 measured at 3.8 seconds. **B** NIBUT to 3rd month measured at 9.5 seconds. **C** Height of tear meniscus (unchanged by IPL treatment). **D** Interferometry (unchanged by IPL treatment). **E** Meibography of the right upper eyelid showing atrophy of the meibomius glands (unchanged by IPL treatment)

The mean difference in OSDI score was − 7.4 _95%_CI [− 13.2; − 1.6] between D0 and 3rd month, − 6.7 _95%_CI [− 12.31; − 1.33] between D0 and D45, and − 9.2 _95%_CI [− 15.32; − 3.57] between D0 and 6th month. The likelihood ratio tests (0.012) and the nonparametric test (0.0035) were significant with maximum improvement in the OSDI score at 6th month (Fig. 2B and 3).

The mean difference in Oxford score was − 0.4 _95%_CI [− 0.65; − 0.16] between D0 and 3rd month, − 0.4 _95%_CI [− 0.64; − 0.19] between D0 and D45, and − 0.4 _95%_CI [− 0.71; − 0.19] between D0 and 6th month. The likelihood ratio tests (0.00021) and the nonparametric test (3*10^-5) were significant with a constant and identical improvement of the Oxford score from D45 to 6th month (Fig. 2C).

The mean difference in interferometry values was − 8.3 _95%_CI [− 14.9; − 1.69] between D0 and 3rd month, − 7.8 _95%_CI [− 14.2; − 1.35] between D0 and D45, and − 6.9 _95%_CI [− 13.9; 0.24] between D0 and 6th month, without significant time influence (non-parametric test was equal to 0.18) (Fig. 2D).

### Standard break-up time, Schirmer test and tear meniscus height do not significantly change after IPL (Table 2)

We did not find significant difference between D0 and 3rd month for the BUT with a mean difference of − 1.35 _95%_CI [− 3.17; 0.47], for the Schirmer test, with a mean difference of − 0.1 _95%_CI [− 2.0; 1.8], nor for the tear meniscus height, with a mean difference of − 0.006 _95%_CI [− 0.4; 0.4] (Table 2)*.* Meiboscores were unchanged. Other data, as meibomian gland loss percentage calculation could not be analyzed due to a lack of reproductibility. Because the analysis area is manually drawn, there may be significant variations from one measurement to another.

**Table 2:** Evolution of the studied parameters during and after IPL treatment between D0 and D45 (end of IPL treatment), D0 and 3rd month and D0 and 6th month. Longitudinal evolution of studied parameters values was assessed using a likelihood ratio test. *P*-values of the non-parametric test are provided between brackets

Parameters	Time	Estimate values	CI 95%	*P* values
NIBUT	Baseline Delta D0 - D45 Delta D0 – M3 Delta D0 – M6	8.9090 seconds	[8.2; 9.7]	*P* value of the likelihood ratio test = 0.0161
		1.1030 seconds	[0.5; 2.1]	
		1.6267 seconds	[0.5; 2.6]	
		1.1265 seconds	[0.5; 2.2]	*P* value of the non-parametric test = 0.0091
OSDI	Baseline D0 - D45 D0 – M3 D0 – M6	44.152	[38.3; 49.6]	*P* value of the likelihood ratio test = 0.0124
		−6.761	[−12.3; −1.3]	
		−7.379	[− 13.2; − 1.6]	
		−9.244	[− 15.3; −3.6]	*P* value of the non-parametric test = 0.0035
Oxford score	Baseline D0 - D45 D0 – M3 D0 – M6	1.600	[−1.4; 1.8]	*P* value of the likelihood ratio test = 0.00021
		−0.4056	[−0.6; −0.2]	
		− 0.4005	[− 0.7; − 0.2]	
		−0.4414	[− 0.7; − 0.2]	*P* value of the non-parametric test = 3*10^-5
Interferometry	Baseline D0 - D45 D0 – M3 D0 – M6	43.133	[37.1; 49.1]	*P* value of the likelihood ratio test = 0.0315
		−7.840	[−14.2; − 1.4]	
		−8.260	[−14.9; − 1.7]	
		−6.880	[−13.9; 0.2]	*P* value of the non-parametric test = 0.1830
BUT	Baseline D0 - D45 D0 – M3 D0 – M6	15.6333	[14.2; 17.2]	*P* value of the likelihood ratio test = 0.0406
		0.8333	[−0.8; 2.5]	
		−1.3503	[−3.2; 0.5]	
		−1.3142	[− 3.2; 0.5]	*P* value of the non-parametric test = 0.0132
Schirmer test	Baseline D0 - D45 D0 – M3 D0 – M6	13.7479	[11.7; 16.1]	*P* value of the likelihood ratio test =0.9764
		−0.3238	[−2.1; 1.5]	
		−0.0746	[−2.0; 1.8]	
		0.1011	[−1.7; 2.1]	*P* value of the non-parametric test = 0.8303
Tear meniscus height	Baseline D0 - D45 D0 – M3 D0 – M6	0.3057	[0.04; 0.6]	*P* value of the likelihood ratio test = 0.4625
		0.2325	[−0.2; 0.6]	
		−0.006	[−0.4; 0.4]	
		−0.0503	[−0.4; 0.3]	*P* value of the non-parametric test = 0.0592

### Safety is excellent

No adverse event was noted during IPL sessions performed with the lowest fluence available on the device (8 J/cm^2^). Only one patient experienced transient redness in the shooting area (redness appeared 1 h after the session and disappeared within a few hours).

## Discussion

Meibomian gland dysfunction (MGD) is a very common cause of ophthalmology consultations. Its multi-daily treatment is very demanding and its effectiveness is very variable. We show here, using objective methods of measurement that IPL delivered by Lacrystim® device can be a non-drug and punctual alternative. Its seems to have a beneficial role on the meibomius glands and thus the lipid layer. The tear film appears to be more stable with an improvement in mucous and lipid layers observed with NIBUT and interferometry measurements and DED symptoms decrease as shown by improvement in OSDI score. Importantly, NIBUT, an objective parameter of tear film stability, improved in parallel with the OSDI score, a subjective parameter, in order to demonstrate DED improvement. The oxford score improvement also reflects a clinical enhancement of DED. However, some parameters remain unchanged after IPL. Standard break-up time is probably not a sensitive test enough. IPL may have no effect on the tear film aqueous phase, evaluated by tear meniscus height, nor on tears quantity, reflected by Schirmer test. Meibomian glands morphology before and after Lacrystim® treatment, although being an important clinical element in the analysis of the lipid layer, could not be assessed due to the lack of reproducibility in the meibographic images analysis. Only initial meiboscore before treatment was determined.

Lacrystim® shows similar results to previously published studies and other IPL devices [23–27]. NIBUT values comparison from one study to another is difficult because analysers use very different image analysis systems. Lacrydiag® detects the very first tear film break-up time whereas other analysers detect an average NIBUT. This explains why we found lower NIBUT values compared to other studies [23, 25, 27, 28]. It is therefore NIBUT evolution before and after treatment that must be taken into account. IPL treatment would improve tear film quality via the lipid layer and decrease DED symptoms. Expression and quality of the meibum appear to improve after treatment. Studied parameters are identical to those of our study [10, 29]: NIBUT or TBUT, interferometry, meibography, OSDI or SPEED (Standard Patient Evaluation of Eye Dryness score) questionnaires. Effect of IPL treatment seems more efficient if it is coupled with manual expression of the meibomius glands [30, 31].

The originality of our work lies in primary endpoint evaluation at 3 months with an extended follow-up over 6 months, unlike most studies that propose a follow-up of only 45 or 75 days [23, 24, 10, 28]. It seemed relevant to measure the primary endpoint at 3 and 6 months to evaluate IPL treatment at a distance from the last treatment session (at D45) and to observe that its effect is maintained over time. Only one study evaluated IPL treatment with a 6-month follow-up [32] and we find comparable results. In our study, treatment with Lacrystim® gave lasting results on NIBUT, OSDI, OXFORD score and interferometry. This raises the question of whether it is possible to carry out an additional treatment session in some patients in case of an early recurrence of the symptoms. Similarly, the relevance of performing an annual treatment session in satisfied patients is debatable.

We performed all the treatments using a fluence of 8 mJ/cm^2^ (the lowest fluence proposed by Lacrystim®) in order to have comparable results between patients and to evaluate the minimal possible effect of the device. The other IPL devices offer higher fluences but without the possibility of treating phototype VI. The Lacrystim® remains currently the only device commercially available that can perform IPL treatment on the darkest phototypes. There is a close relationship between skin type and the energy used in IPL treatment: lower energy is preferred for darker skin types and higher energy for lighter skin types, although the optimal fluence for each phototype is not determined. As the majority of our patients were phototypes III and IV, a fluence of 8 J / cm2 seems relevant to us without the risk of over or under treatment.

IPL appears to be a safe treatment. We found no adverse event or complication in our cohort. Few complications have been published. Rare cases of uveitis [33] and one case of choroidal neo-vascularization [34] have been reported. This shows the importance of wearing protective glasses during the procedure [35] and the need to strictly position the shots on the cheekbone under the lower eyelids.

However, there are methodological limitations in most studies, including our own. Due to the retrospective design and control group absence, this study is exploratory and preliminary. The results cannot be extrapolated and a randomized controlled trial (RCT) on a larger number of patients is needed to confirm the relevance of Lacrystim®. Nevertheless, this work allows to present first indicative results on the use in practice of this new IPL device. To date, there are very few prospective, controlled, RCT published on this topic [31, 28, 36, 32, 37]. It is important but difficult to select an objective and robust clinical primary endpoint [22]. We choose NIBUT as a primary endpoint because it is a valid and objective evaluation criteria [14] which reproductible and automatic measurement by Lacrydiag® limits the placebo confounding factor in our study. Indeed, in absence of a control group and double-mask, the placebo effect remains important in pathology such as DED. This is a common pitfall found in the literature [38].

Similarly, considering the heterogeneity of patients with MGD, the study of the effect of IPL on specific subgroups of patients seems interesting: for example, severe MGD during graft-versus-host disease, contact lens wearers with MGD causing an excessive lens fouling or MGD during vernal keratoconjunctivitis. This type of treatment in the pediatric population should be investigated in addition to palpebral massages, which are difficult to perform in practice in children.

## Conclusion

Treatment of MGD using IPL delivered by the Lacrystim® device seems effective, fast and safe. Its coupling with the Lacrydiag® diagnostic device is interesting and allows an objective evaluation of tear film parameters. A RCT is needed to confirm and validate these preliminary results.

## Data Availability

Data that support the findings of this study are not openly available and are available from the corresponding author upon reasonable request.
